# Broadband absorption using all-graphene grating-coupled nanoparticles on a reflector

**DOI:** 10.1038/s41598-020-76037-x

**Published:** 2020-11-04

**Authors:** Shiva Hayati Raad, Zahra Atlasbaf, Carlos J. Zapata-Rodríguez

**Affiliations:** 1 Department of Electrical and Computer Engineering, Tarbiat Modares University, Tehran, Iran; 2 Department of Optics and Optometry and Vision Science, University of Valencia, 46100 Burjassot, Spain

**Keywords:** Nanophotonics and plasmonics, Optical materials and structures

## Abstract

In this paper, the hybridized localized surface plasmon resonances (LSPRs) of a periodic assembly of graphene-wrapped nanoparticles are used to design a nanoparticle assisted optical absorber. Bandwidth enhancement of this structure via providing multiple types of plasmonic resonances in the associated unit cell using two densely packed crossly stacked graphene strips is proposed. The designed graphene strips support fundamental propagating surface plasmons on the ribbons, and gap plasmons in the cavity constructed by the adjacent sections. Graphene strips exhibit a hyperbolic dispersion region in the operating spectrum and assist in the bandwidth enhancement. Moreover, since the nanoparticles are deposited on the top strips, real-time biasing of them can be easily conducted by exciting the surface plasmons of the strip without the necessity to electrically connect the adjacent nanoparticles. The overall dynamic bandwidth of the structure, using a two-state biasing scheme, covers the frequencies of 18.16–40.47 THz with 90% efficiency. Due to the symmetry of the structure, the device performs similarly for both transverse electric (TE) and transverse magnetic (TM) waves and it has a high broadband absorption rate regarding different incident angles up to 40°. Due to the presence of 2D graphene material and also using hollow spherical particles, our proposed absorber is also lightweight and it is suitable for novel compact optoelectronic devices due to its sub-wavelength dimensions.

## Introduction

Nanoparticle assisted optical absorber design is a well-known approach for manipulating the interaction between the electromagnetic wave and matter at infrared and optical frequencies^1,2^. As an instance, simple cubic patterns of gold cylindrical and spherical particles have been proposed to convert the laser energy into heat in non-destructive testing and medical diagnostic applications^3^. Moreover, an optical meta-surface constructed by silver ellipsoidal particles behaves like a resistive sheet and by engineering its surface impedance, a Salisbury absorber can be designed^4^. These narrowband absorbers can be possibly used in light filters or digital sensors^5^.

An inherent drawback of metal-based nanoparticle assisted absorbers is the formation of an oxide shell on the surface of the nanoparticle due to contact with air or moisture. This layer may significantly affect the scattering pattern and should be taken into account in practical applications^6^. Graphene, a two-dimensional nanomaterial, is highly resistant to oxygen. Besides, due to being chemically inert and strongly transparent to light, it is a promising candidate in various novel optical devices^7^. Considering the graphene-wrapped structures, free-standing spherical nanoparticles behave as a reconfigurable reflector away from their plasmonic resonances^8^. Moreover, by dispersion engineering of the resonance channels provided with the plasmonic resonances of a double graphene layer spherical particle, a superscatterer can be designed^9^. Enhanced energy transfer and giant near field enhancement are two other examples exhibiting the wide range of applications provided by localized plasmons in graphene-coated particles^10,11^.

Specifically, graphene-based perfect metamaterial absorbers have attracted a surge of research interest due to the extraordinary electronic and photonic properties of graphene material^12^. Focusing on graphene-wrapped structures, enhanced absorption is reported in the sub-wavelength graphene-coated wires^13^. Moreover, by preparing the graphene-coated particles on top of an optical mirror, the plasmonic resonances result in the narrow-band perfect tunable optical absorption, originating from the large extinction cross-section of an isolated particle concerning its geometrical cross-section^14,15^.

Generally, bandwidth enhancement in the resonant structures can be achieved by providing various closely packed resonances, exploiting multi-resonance unit cells, multi-layered structures, and multiple-order resonances^16–19^. For instance, broadband absorption in graphene-wrapped fractal oligomers is the result of plasmonic resonances of various orders arising from plasmonic hybridization in the top, bottom, and lateral surfaces^19^, revealing that the combination of the two of the aforementioned methods is used. Bandwidth enhancement of optical absorption in graphene-based devices can also be achieved by combining graphene with various photonic structures, such as dielectric waveguides, photonic crystals, plasmonic metamaterials, dielectric metamaterials, integrated microcavities, and other types of resonant structures^20,21^. Hyperbolic metamaterials (HMM), being anisotropic artificial materials, are other alternatives that have been widely used in manipulating optical absorption^22,23^. In this regard, broadband absorption using tapered HMM array is experimentally realized in the visible and infrared ranges^24^. In another research, ultra-broadband light absorption based on photonic topological transitions in HMMs is proposed^25^. Moreover, a polarization-dependent broadband infrared absorption inside the hyperbolic frequency region is attained using α-phase molybdenum trioxide (MoO_3_) nanostructures^26^.

Hyperbolic metamaterials are commonly designed by stacking alternating metal and dielectric layers or using a lattice of metallic nanowires embedded in a dielectric matrix^27^. Stacked graphene-dielectric pairs can be used to realize gate tunable hyperbolic metamaterials to be possibly used as a super-absorber^28^. Hyperbolic meta-surfaces based on nanostructured van der Waals materials provide opportunities for the realization of subwavelength-scale structures^29^. Hexagonal boron nitride (hBN) and borophene are examples of the two-dimensional Van der Waals natural hyperbolic materials, respectively used in the design of the multi-band and single band absorbers^30,31^, and both of them are lacking the gate tunability feature. The hyperbolic plasmons of densely packed graphene strips enable voltage reconfigurable flatten optics in a broad bandwidth^32^ and it is of interest in this research.

To design a broadband absorber, we have exploited the multi-resonance, multi-layered, and multi-ordered schemes simultaneously by using hollow graphene-wrapped particles on top of two densely packed crossly stacked graphene strips. It is shown that the hybridized localized surface plasmon resonances in the graphene-coated spherical particles result in the excitation of quadrupole modes that cover the high frequencies of the desired spectrum. These resonances are manipulated with the propagating surface plasmons and gap plasmons of densely packed dual graphene ribbons to enhance the operating bandwidth. The reason for choosing the densely packed strips as the other resonating element of the unit cell is its capability to support hyperbolic plasmons.

Our design offers polarization-insensitive optical response due to the crossly stacked strips and it is lightweight due to the use of 2D graphene material and hollow spherical particles. Another feature of our proposed structure is offering a simple biasing scheme. In general, graphene-based devices with discontinuous elements suffer from the fabrication complexity due to the requirement for properly designed biasing networks^33^. Grating-based graphene structures can be simply biased with a cathode in the form of a bridge^34^ and real-time biasing of each ribbon is also feasible by using field-programmable gate array (FPGA) hardware^35^. Therefore, in the proposed design, the LSPRs in an assembly of isolated spherical nanoparticles are excited by residing them on top of the graphene strips. Note that exciting the localized surface plasmons via propagating surface plasmons is a well-known approach that was proposed for the metallic nanoparticles before^36^ and our design benefits the same strategy for a graphene-based structure.

As a final comment, graphene material can be prepared by chemical vapor deposition (CVD) or by epitaxial growth on silicon carbide or metals^37^. Moreover, as a result of the van der Waals force, graphene can be wrapped around particles of various shapes and sizes^38^. For instance, graphene oxide wrapped gold nanoparticles have been fabricated by a one-step synthesis^39^. Densely packed graphene strips can be fabricated by electron beam lithography^40^. The plasmonic grating with gold nanoparticles resided on its surface is realized by respectively fabricating the sections with the colloidal self-assembly and self-assembly techniques^41^. Alternatively, electrohydrodynamic (EHD)-jet printing is proposed to attain a nanoparticle assembly on the electrostatically attractive surface^42^. Considering the above-mentioned techniques, our proposed structure is also experimentally realizable with current nanofabrication technology.

The paper is organized as follows. Initially, the optical performance of densely packed graphene strips and graphene-coated spherical particles are disclosed. Later, dual graphene ribbons are hybridized with the graphene-based meta-surface constructed by the spherical particles for bandwidth enhancement. Due to the contact of particles and top strips, the dynamic biasing network can be easily implemented through optical bridges, connecting the strips. It is worth noting that a typical approach to design the metamaterial plasmonic light trapping devices is solving Maxwell’s equations via computational electromagnetics such as the characteristic matrix method, finite element method, and circuit design, to name a few^43,44^. The analytical investigation of our proposed structure is complicated due to the tensorial surface conductivity required for the appropriate modeling of the equivalent surface conductivity of graphene strips^45^. Therefore, we will focus on the numerical analysis of this structure.

## The optical performance of densely packed graphene strips and graphene-coated spherical particles

In this section, the optical performance of densely packed graphene strips and graphene-wrapped spherical particles are discussed. These two elements will be used in the unit cell of our proposed broadband absorber in the next section. Matlab-based programs are used to investigate the effective surface conductivity of graphene-based strips and the extinction efficiency of graphene-coated nanoparticles. The results of these subsections will aid to opt suitable initial values for the optical and geometrical parameters.

### Optical performance of densely packed graphene strips

**Figure 1:**
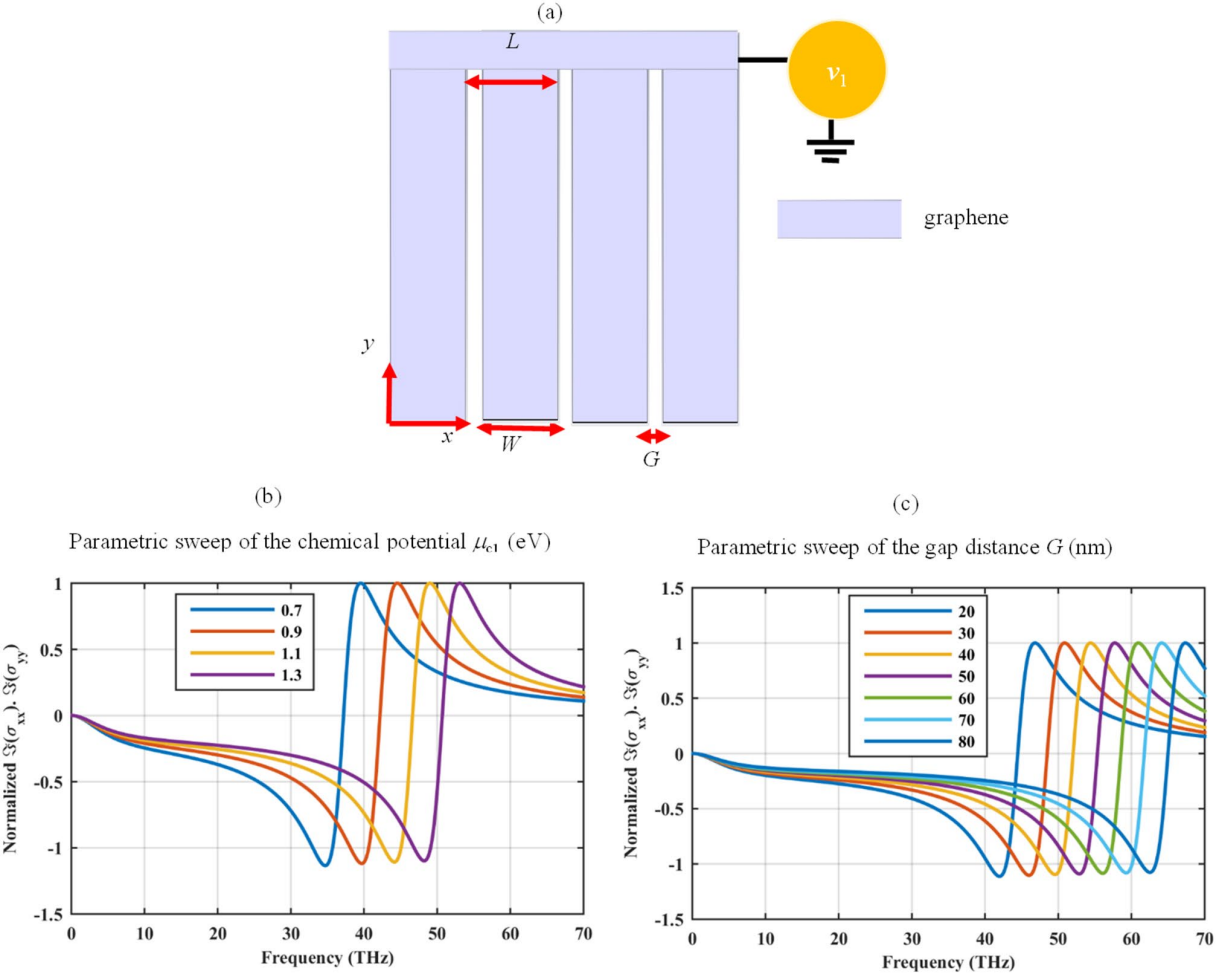
(**a**) Free-standing densely packed graphene strips biased with a bridge using the bias voltage *v*_1_. Normalized $\,\Im \left( {\sigma_{xx} } \right).\Im \left( {\sigma_{yy} } \right)$ for *W* = 220 nm (**b**) considering different chemical potentials *μ*_*c*1_ (eV) for *G* = 20 nm and (**c**) considering different gap distances *G* (nm) for *μ*_c1_ = 1 eV. The scattering rate is 20 meV in all simulations. The closed-form formulas for finding the required bias voltage for each chemical potential can be found in^48^. The symbol $\Im$ represents the imaginary part of the complex function and the normalization factor is the maximum value of $\Im \left( {\sigma_{xx} } \right).\Im \left( {\sigma_{yy} } \right)$.

Let us consider the free-standing densely packed 2D graphene strips of Fig. 1a with the width of *W* and periodicity of *L*. The strips can be biased through a bridge using electrostatic bias voltage *v*_1_. The influence of the thin bridge is neglected throughout the paper. This structure behaves as a uniaxial meta-surface and it supports a hyperbolic dispersion region enabling the flattened optics^40^. To benefit the hybridization of the hyperbolic plasmons with the LSPRs, it is essential to extract the hyperbolic dispersion spectrum of the graphene strips considering various geometrical and optical parameters. The isotropic surface conductivity of the continuous graphene sheet $\sigma$ in the absence of magnetic bias, for the moderate and low frequencies $\left( {\hbar \omega < \mu_{c} } \right)$, and large doping $\left( {\mu_{c} \gg k_{B} T} \right)$, can be approximated as^46^:

1 $$ \sigma \left( \omega \right) \approx \frac{{ie^{2} \mu_{c} }}{{\pi \hbar^{2} \left( {\omega + i\Gamma } \right)}} $$

where $e$, $k_{B}$, $\hbar$, and *T* are respectively the electron charge, Boltzmann’s constant, reduced Planck’s constant, and temperature. Moreover, $\Gamma$ stands for the charge carriers scattering rate and it is considered to be 20 meV (corresponding to the relaxation time of *τ* = 32.9 fs)^47^, throughout this paper. The applied bias voltage alters the graphene chemical potential *μ*_*c*_ and these two parameters can be related via approximate analytical equations^48^. Note that the chemical potential as high as 2 eV can be obtained experimentally^49^. The elements of the uniaxial surface conductivity of the densely packed graphene strips are^50^:

2 $$ \sigma_{xx} = \frac{{W\sigma \sigma_{C} }}{{L\sigma_{C} + W\sigma }}\quad \sigma_{yy} = \sigma \frac{W}{L}\quad \sigma_{xy} = \sigma_{yx} = 0 $$

where $\sigma_{C} = - i2\omega \varepsilon_{0} \varepsilon_{eff} \left( {{L \mathord{\left/ {\vphantom {L \pi }} \right. \kern-\nulldelimiterspace} \pi }} \right)\ln \left[ {\csc \left( {{{\pi G} \mathord{\left/ {\vphantom {{\pi G} {2L}}} \right. \kern-\nulldelimiterspace} {2L}}} \right)} \right]$ is the effective grid conductivity, *ε*_*eff*_ is the effective permittivity of the substrate, and *G* is the gap distance between two adjacent strips. The hyperbolic dispersion region covers the spectrum in which the imaginary parts of the diagonal surface conductivities have opposite signs. To investigate this condition, Fig. 1b illustrates the normalized value of $\,\Im \left( {\sigma_{xx} } \right).\Im \left( {\sigma_{yy} } \right)$ for the strips with *W* = 220 nm, *L* = 240 nm, and considering various chemical potentials. The symbol $\Im$ represents the imaginary part of the complex function and the normalization factor is the maximum value of $\Im \left( {\sigma_{xx} } \right).\Im \left( {\sigma_{yy} } \right)$. Note that finite-size quantum effects can be neglected for the strip widths greater than 10 nm^51^. Based on Fig. 1b, flattened hyperbolic material with tunable transition frequency at the infrared spectrum can be attained using the densely packed graphene strips with the above-mentioned design parameters^50^. The hyperbolic region can be further manipulated by varying the parameter *G*. In Fig. 1c, the gap distance is varied from 30 to 80 nm for the fixed periodicity of *L* = 240 nm, and the chemical potential of *μ*_*c1*_ = 1 eV. Increased gap distances lead to the blue shift of the transition frequency.

### Optical performance of graphene-wrapped spherical particles

**Figure 2:**
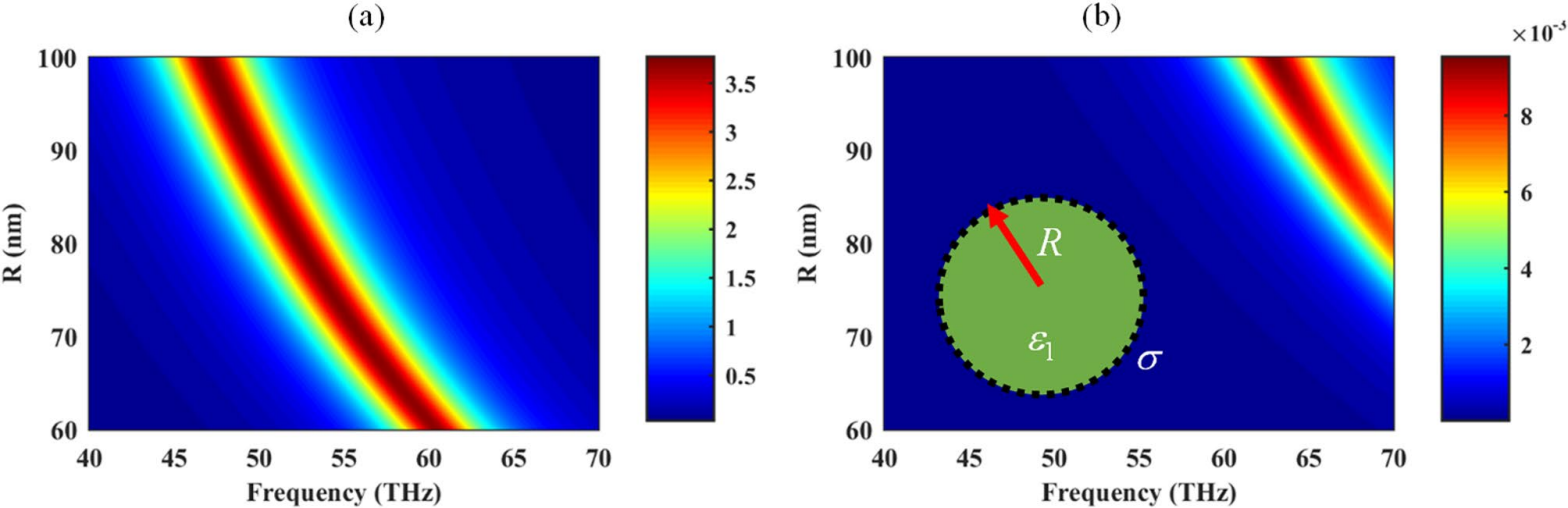
The extinction efficiency of (**a**) dipole and (**b**) quadrupole modes for hollow (*ε*_1_ = 1) graphene-wrapped spherical particle with various radii *R*. The optical parameters of graphene cover with the surface conductivity *σ* are *τ*  = 32.9 fs, and *μ*_*c*1_ = 0.95 eV. The extinction efficiency is the extinction cross-section obtained by the modified Mie-Lorenz theory, normalized to the geometrical cross-section^52^.

To get some insight into the localized surface plasmon resonances of the graphene-coated spherical particles, an isolated particle under plane wave illumination, as shown in the inset of Fig. 2, is considered. The normalized extinction efficiency for hollow spheres (*ε*_1_ = 1) with the radii *R* ranging from 70 to 100 nm, the relaxation time* τ*  = 32.9 fs, and *μ*_*c*_ = 0.95 eV is illustrated in Fig. 2 based on modified Mie-Lorenz theory considering both dipole and quadrupole modes^52^. The normalization factor is the geometrical cross-section of the spheres. In general, increasing the sphere radius red-shifts the resonance frequency. Moreover, the higher-order modes are not strongly excited in the isolated spheres, but, they play a crucial role in the performance of the assemblies of these particles, as will be discussed later.

**Figure 3:**
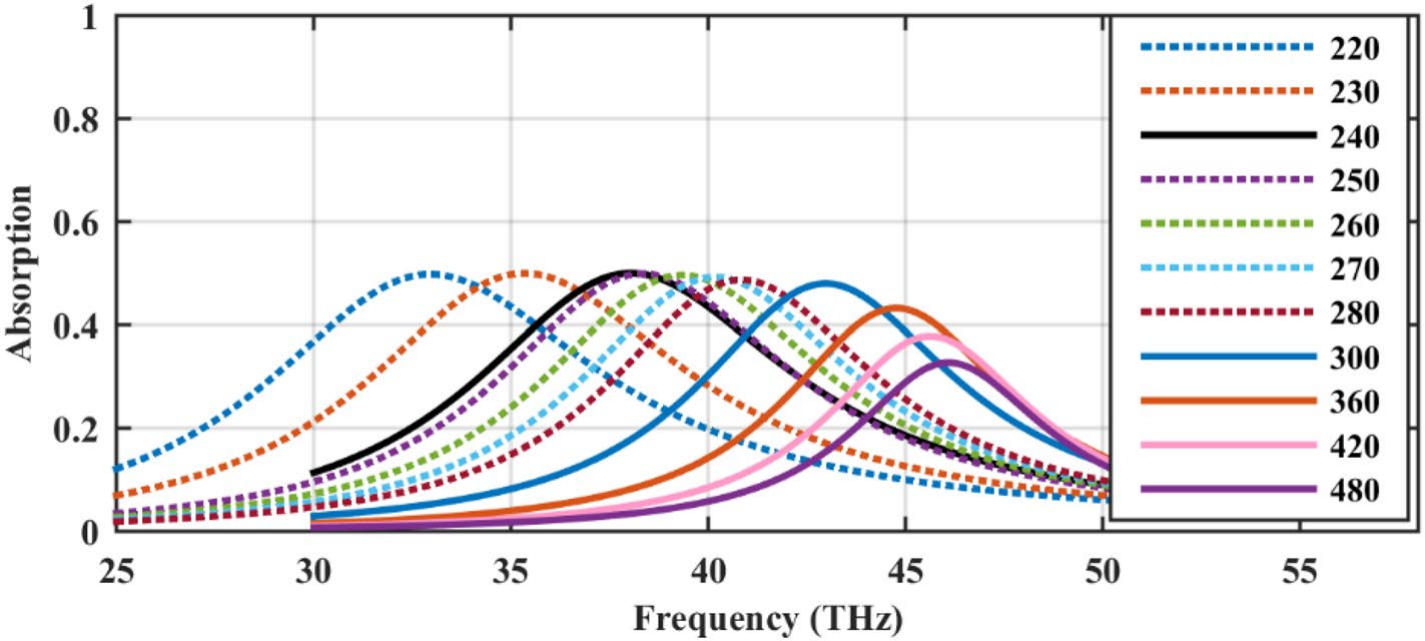
The absorption rate of the free-standing graphene-wrapped particles in a square lattice for different periodicities (nm). The other parameters of the free-standing hollow spheres are *R* = 100 nm, *τ* = 32.9 fs, and *μ*_*c*1_ = 0.95 eV. Note that to obtain this figure, the graphene-coated particle in the inset of Fig. 2 is considered in the unit cell analysis of CST 2017 software by considering various gaps between the adjacent spheres.

Regarding the optical absorption of free-standing sphere array with the radii *R* = 100 nm and optical parameters of *τ* = 32.9 fs, and *μ*_*c*1_ = 0.95 eV for different values of the periodicity, as in Fig. 3, it is clear that dense arrays make red-shift of the Mie resonance. Also, for an interval of periodicities (220–280 nm), the 50% absorption rate, which is the maximum value for any free-standing symmetric array^53^, is attained. The impact of periodicity in this interval is to set the frequency peak. Note that although particles with sub-nm gap distances are experimentally realizable^54^, we have chosen the minimum distance as 20 nm to prevent the proximity and non-local effects^55^. Note that the value of the chemical potential matches with the optimized value of the next section in the final design.

In Ref.^47^, simple expressions to evaluate the complex frequencies characterizing the extinction resonances of a single graphene sphere is provided by calculating the poles of the modified Mie–Lorenz coefficients, where its real part gives the peak center and its imaginary part denote its full width at half maximum (FWHM), approximately^38^. Noting that sparse arrays resemble the isolated spheres, once the particles are arranged in an array, a spectral broadening is evident. Both redshift and spectral broadening are produced by coupling effects between spheres in the array. In a dense 2D array of plasmonic sub-wavelength spheres, as the sphere radius increases or as the lattice constant decreases, i.e., as the coverage increases, the parallel mode resonance is shifted towards lower frequencies and the normal mode resonance to higher frequencies^56^. Since the normal-incidence for the illuminating plane wave is considered here, only parallel mode is present in our simulations and its performance is in agreement with^56^. Note that this figure is produced using CST 2017 software package and the simulation setup will be introduced in the next section in detail.

Based on the results of the previous two subsections, by wisely choosing the optical and geometrical parameters of the graphene-wrapped spherical particles^47^, the associated localized surface plasmon resonances will lie on the hyperbolic dispersion region of the densely packed strips leading to an efficient wideband enhanced absorption.

## Broadband absorber design using graphene-based grating-coupled nanoparticles

In this section, broadband absorber design using graphene-based grating-coupled nanoparticles will be presented. The unit cell consists of a graphene-coated hollow spherical particle resided on top of the two densely packed crossly stacked graphene strips. The necessity of each element for the broadband response is clarified and excited surface plasmons are exhibited by providing the spatial distribution of the electric field. Finally, various parametric studies are conducted to discuss the influence of the geometrical parameters on broadband optical absorption.

### Broadband absorber design using multiple resonances in the unit cell

**Figure 4:**
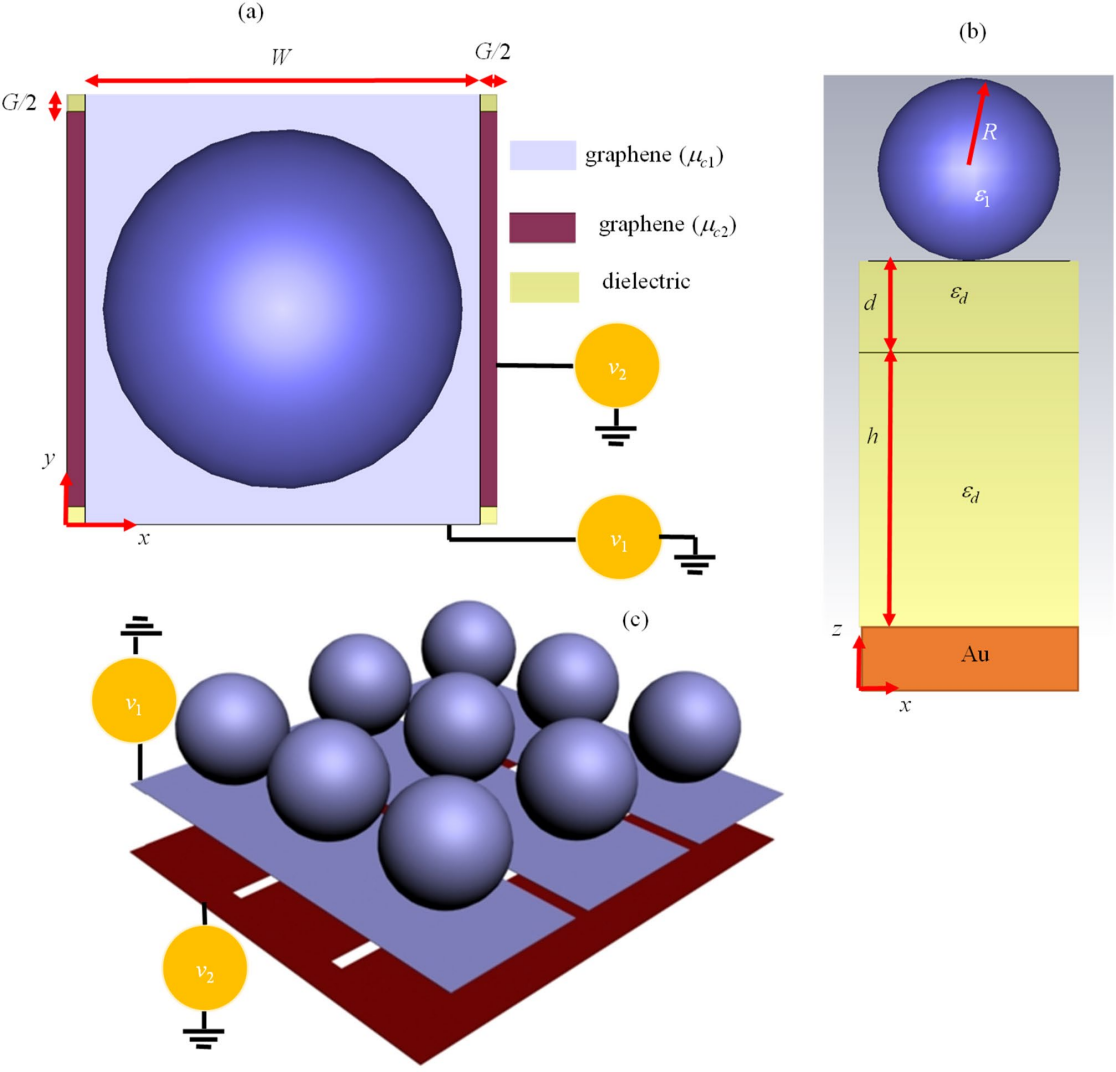
(**a**) Top and (**b**) side views of graphene-based grating-coupled spherical nanoparticles on top of the metallic mirror. The biasing bridges are not illustrated in these sub-figures. (**c**) 3D view of our proposed wideband absorber which is biased with the bridges. For better illustration, the supporting dielectric is not shown in this sub-figure.

In the following paragraphs, the previously introduced 2D hyperbolic meta-surface will be used as a substrate for the broadband absorber design. The unit cell of our proposed wideband absorber which is constructed by two stacked densely packed graphene strips with the width of *W* = 220 nm and gap distance of *G* = 20 nm is illustrated in Fig. 4. Graphene-coated hollow spherical nanoparticles with the radius of *R* = 100 nm have prepared on the top of the 2D hyperbolic meta-surface. Therefore, by exciting the propagating surface plasmons of the strips, the localized surface plasmons of the particles are excited due to electrical connection. The relative distance between the two strips is *d* = 100 nm and the lower one is rotated 90° concerning the other one. The reason for this modification relies on the fact that even-layered crossly stacked anisotropic 2D material nano-structures support complementarily excited surface plasmon resonances in the two lattice directions and are capable of polarization-independent absorption^57^. For simplicity, the geometrical parameters of both strips are considered to be identical. The supporting substrate is a dielectric with the height of *h* = 1900 nm and relative permittivity of *ε*_*d*_ = 2^58^. There is also a metallic mirror beneath the substrate to contribute to the absorption enhancement by suppressing the transmitted wave^59^. This layer is constructed by gold (Au) material with the dispersive constitutive parameters extracted from^60^. This layer is also beneficial in the electrostatic biasing of the device as a reference plane^61^. The 3D view of the absorber is exhibited in Fig. 4c in which the ribbons of each layer are connected through bridges for the ease of practical applications^34^. The subsequent results are provided with the unit cell analysis of the frequency domain solver of CST 2017 commercial software package. In all simulations, the first TE and TM modes are considered to be excited in the Floquet ports, unless otherwise is stated.

**Figure 5:**
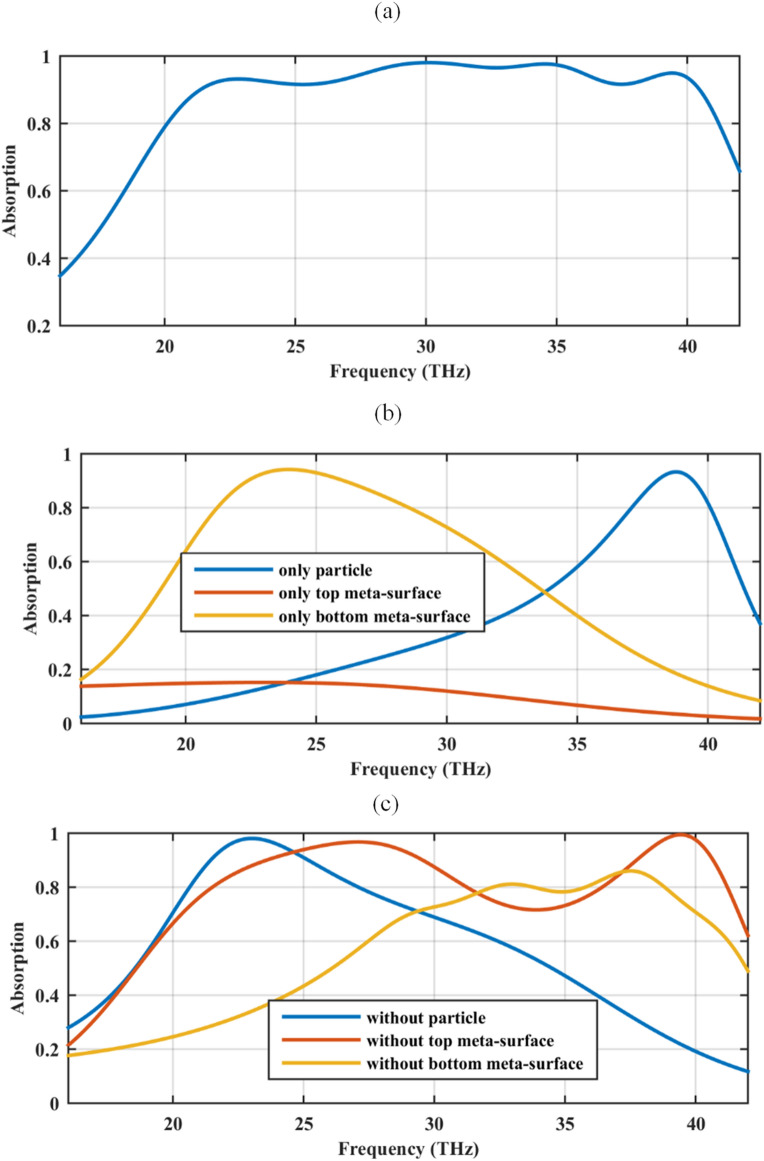
(**a**) The absorption spectrum of our proposed absorber in Fig. 4 for the chemical potential set (0.95,1.4) (eV), (**b**) considering each graphene-based element of the unit cell individually and (**c**) in three cases, each containing only two of the graphene-based sections. The graphene-coated spheres are hollow and the relative permittivity of the substrate is 2. The geometrical parameters are *W* = 220 nm, *G* = 20 nm, *R* = 100 nm, *d* = 100 nm, and *h* = 1900 nm.

The absorption spectrums of our proposed structure for a set of chemical potentials (*μ*_*c*1_, *μ*_*c*2_) (eV) which are associated with the bias voltages (*v*_1_,*v*_2_) (V) is illustrated in Fig. 5a. The figure corresponds to the set of (0.95, 1.4) eV which is obtained by optimization through various parametric sweeps. The broadband absorption with the 90% absorption rate for the frequency span ranging from 21.36–40.52 THz is confirmed. The covering bandwidth is comparable with that of Ref.^58^. The advantage of our proposed structure is using a much smaller unit cell. The periodicity in Ref.^58^ is 10 μm while in our design it is 240 nm. Similar operating wide bandwidths can be also found in Refs.^62,63^, respectively designed with four-ring elements and tapered patch antennas. Note that wideband absorbers based on graphene sheet or pattern are mainly designed in the terahertz frequencies and lower^64–66^. The dynamic bandwidth of the proposed absorber will be compared with graphene-based particle assisted absorbers in the last section. It should be emphasized that loosely coupled graphene strips behave as RLC resonators and enables the optical absorption at resonance^67^. In the presented simulations, graphene isotropic local surface conductivity is applied to the densely packed strips. The procedure of extracting the anisotropic effective surface conductivity of hyperbolic meta-surfaces using commercial software packages can be found in^68^.

To show the necessity of each graphene-based section, in Fig. 5b, the absorption rate of each individual graphene-based section is illustrated. Figure 5b indicates that spheres play the main role for the highest frequencies, whereas bottom strips do it for the lowest frequencies. The top strip plays a minor role when isolated because ribbons are parallel to the incident electric field, preventing the excitation of surface plasmons in this sheet.

For further clarification of the performance, the absorption spectrum of three geometries obtained by removing one of the designed layers each time is included in Fig. 5c. Specifically, the blue curve is associated with the optical absorber constructed by crossly stacked double graphene strips. Moreover, red and yellow curves are respectively obtained by considering only the bottom and top strip in the simulation while maintaining the spherical particle. When combining spheres and top strip, scattered fields from spheres enable the excitation of surface plasmons at mid frequencies propagating in the top strip, thus increasing the absorption at such mid-range of frequencies. This effect is caused by the near-field coupling of the sphere and the top strip via gap plasmons. This can be inferred when comparing Fig. 5b (only spheres and only top strip) and Fig. 5c (without bottom strip). In conclusion, the coupling between the sphere array and top strip plays an important role. Coupling between the top and bottom strip sheets is very weak, practically negligible, as can be inferred when comparing Fig. 5b (only bottom strip) with Fig. 5c (without particles), which are very similar, almost identical. This conclusion can be also sustained by the fact that the response of the whole setup almost is invariant to the position of the bottom strip, as will be further investigated in the next sub-section.

Another outstanding feature of our proposed absorber is using the graphene-wrapped hollow particles in the design. Core material impacts the absorption amount of graphene-wrapped spheres since its decrease is equivalent to the decrease in the geometrical size of the system resulting in blue shift and enhancement of absorption efficiency^69^. Considering hollow particles has another advantage and that is providing a lightweight device. The fabrication fellow of hollow graphene-wrapped particles can be summarized as follows. After preparation of the graphene sheet and silicon dioxide (SiO_2_) nanoparticles, they should be mixed by ultrasonic dispersion in the deionized water, then dried by vacuum freeze-drying process. After annealing the resulted powder in argon gas, the SiO_2_ template can be removed by immersing 20% hydrofluoric acid (HF) solution with stirring^70^.

**Figure 6:**
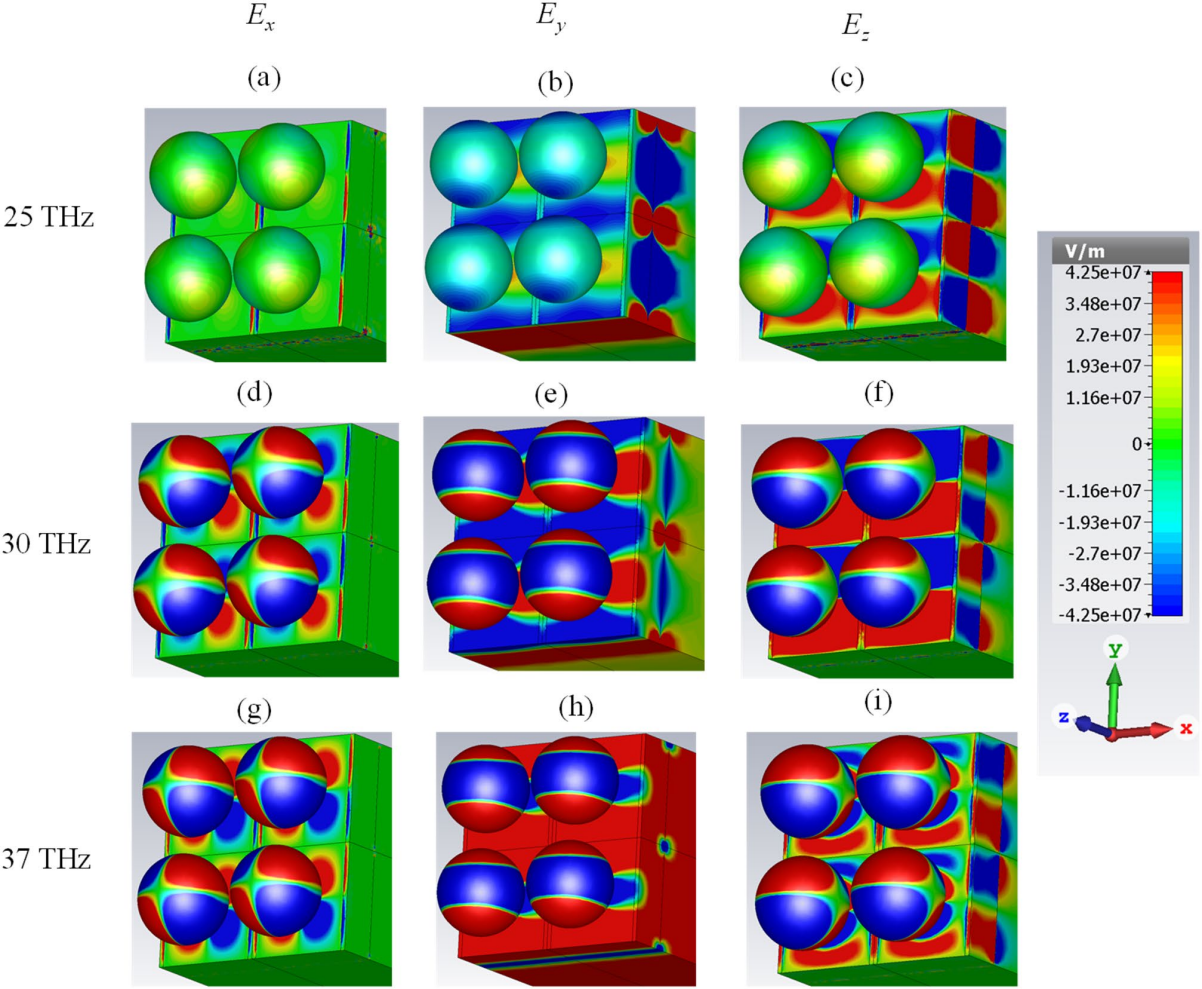
The electric field distributions at three frequencies for the absorber with the chemical potential set of (0.95, 1.4) eV (**a**–**c**) 25 THz (**d**–**f**) 30 THz and (**g**–**i**) 37 THz. The graphene-coated spheres are hollow and the relative permittivity of the substrate is 2. The geometrical parameters are *W* = 220 nm, *G* = 20 nm, *R* = 100 nm, *d* = 100 nm, and *h* = 1900 nm.

To further investigate the performance, the electric field distributions at three frequencies are illustrated in Fig. 6 for all spatial directions. Based on the figure, the quadrupole localized surface plasmons of the spherical particles with different degrees are excited in Fig. 6d–i. In the analytical investigation, they are attained by varying the degrees of the associated Legendre functions in the modified Mie–Lorenz solution^47^. For the sake of clarity, the surface charge distributions which are proportional to $P_{l}^{n} \left( {\cos \theta } \right)e^{in\phi }$ are regenerated from Ref.^47^ for the quadrupole modes (*l* = 2) of various degrees (*n* = 0, 1, 2). The symbol $P_{l}^{n}$ represents the associated Legendre function and the results are summarized in Table 1. Note that we plot the real part of the latter expression and that negative *n* values differ from their positive counterparts only by a rotation. It should be noted that higher-order plasmonic resonances are also capable of efficient energy absorption^71^ and they are excited due to the hybridization^72^.

**Table 1:**
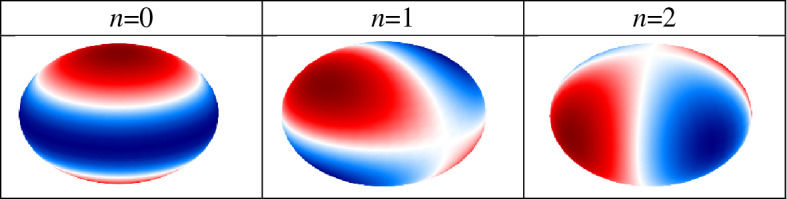
The surface charge distributions for the quadrupole mode (*l* = 2) with various degrees (*n*) regenerated from^47^.

The excited plasmons on the sub-wavelength strip gaps are illustrated in Fig. 6a,d,g. Enhanced electric field distributions at the gap can be interpreted as different guided mode excitations originating from the coupling of air gaps and paired plasmonic nanostructures^73^. Moreover, gap plasmons occurring between a given sphere and the graphene strip set below, enable plasmonic coupling in the form of gap plasmons with increased absorption. Gap plasmons exhibit the concentration and thus the absorption of the confined fields due to the large effective index^74,75^. These resonances appear only for the sufficiently narrow inter-element gaps in which highly coupled plasmons result in a metal–insulator–metal (MIM) like performance supporting a cavity mode or plasmonic standing wave, and they are characterized by strong absorption of the incident light^76,77^. Note that excitation of gap plasmons have been reported using a variety of structures including but not limited to a Bottle-like narrowband absorber, MIM structures with nanometer-sized spacers, deep metallic grating with narrow slits, and paired nanowire waveguides^78–81^.

Another efficient resonance for the absorption is the propagating surface plasmons in the stacked strips. This particular mode in the strips shows an antinode (max/min) at the center of the strip for *E*_*y*_ in Fig. 6b,e. Moreover, it shows a node (zero) at the center of the strip for *E*_*z*_ in Fig. 6c,f,i. This is compatible with an edge mode, and also with a bulk plasmon mode (in the latter case, the fundamental mode), as can be inferred when comparing the field with those of Refs.^82,83^. Our proposed structure can be considered as a substrate mediated plasmonic hybridization technique similar to the graphene decorated silver nanoparticles, metallic nanoparticle-dimer on a mirror, and nanoparticle/graphene/film combinations^84–86^.

**Figure 7:**
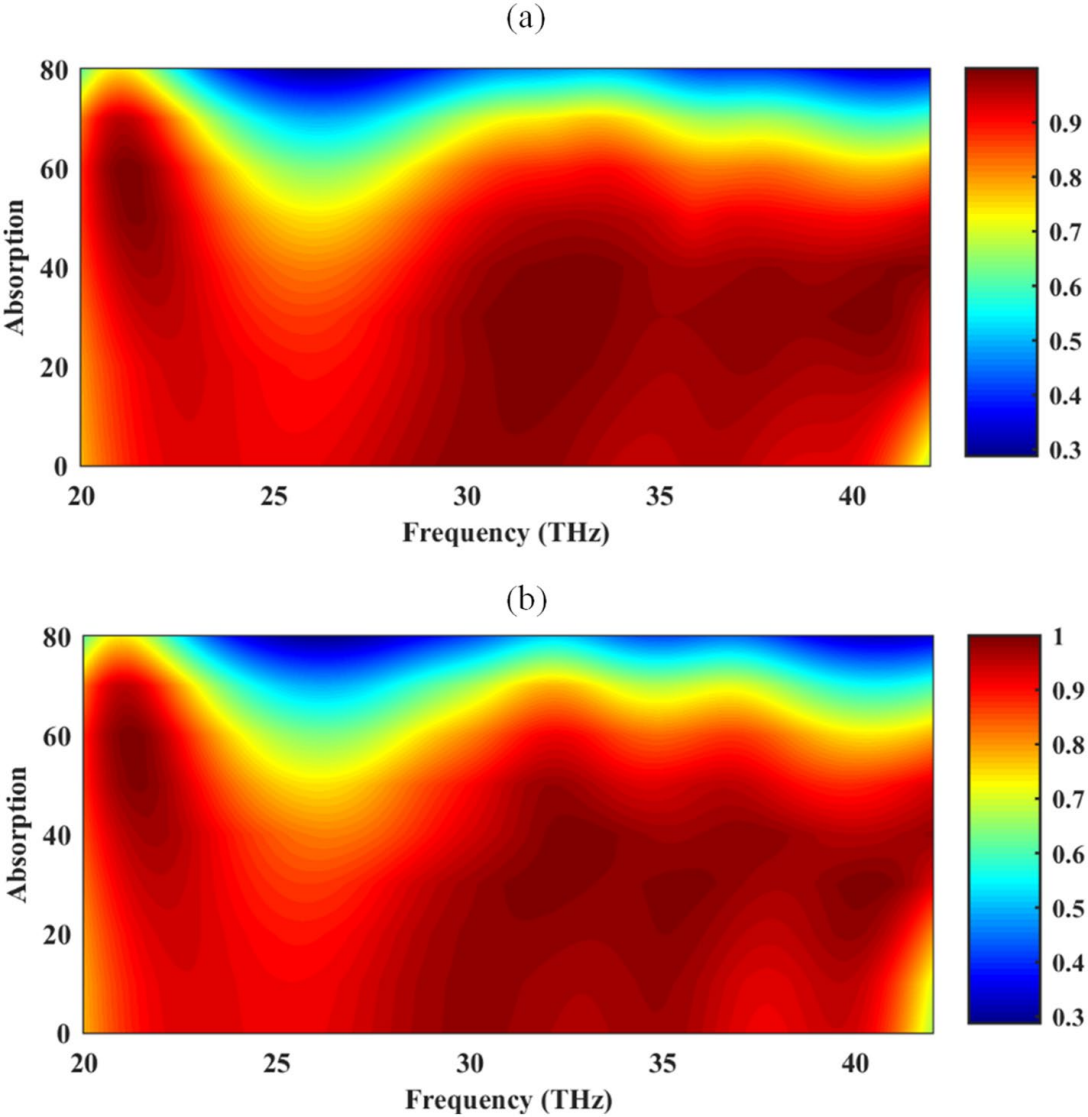
The absorption spectrum of the proposed structure in Fig. 4 for (**a**) TE and (**b**) TM modes for oblique incidence considering the chemical potential set of (0.95, 1.4) eV for biasing. The graphene-coated spheres are hollow and the relative permittivity of the substrate is 2. The geometrical parameters are *W* = 220 nm, *G* = 20 nm, *R* = 100 nm, *d* = 100 nm, and *h* = 1900 nm.

To investigate the influence of the polarization and incident angle of the electromagnetic source on the amount of absorption, the incident angle *θ* is varied from 0° to 80° for TE and TM polarizations. The simulation results, illustrated in Fig. 7, confirms that high absorption is achievable for both TE and TM waves under any incident angle up to 40°. This feature is attained thanks to the fourfold rotational structural symmetry of the proposed unit cell. It should be emphasized that in the proposed design, orthogonally stacked graphene nano-ribbon pairs are the key to achieve polarization independence performance similar to the structures constructed by other 2D anisotropic materials^57,87^. The minor difference in the performance of TE and TM modes is attributed to residing graphene-wrapped particles on the top strip.

### Parametric study of geometrical factors

**Figure 8:**
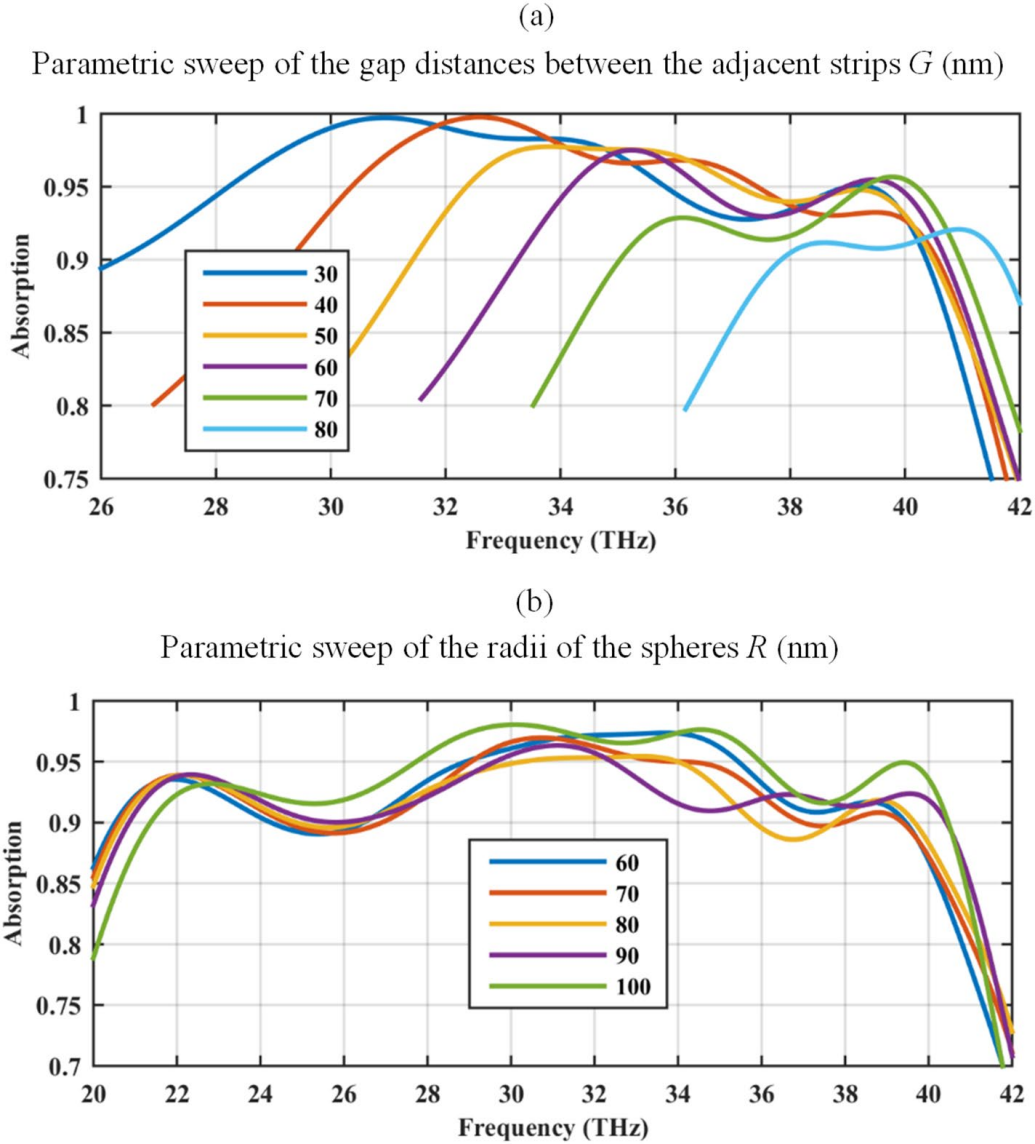
The influence of the geometrical parameters on the optical performance, (**a**) gap distance of the strips *G* (nm), and (**b**) radius of the sphere *R* (nm). The periodicity of 240 nm is maintained in both cases. The graphene-coated spheres are hollow and the relative permittivity of the substrate is 2. The initial geometrical parameters are *W* = 220 nm, *G* = 20 nm, *R* = 100 nm, *d* = 100 nm, and *h* = 1900 nm.

The impact of the geometrical parameters on the optical performance of the absorber is investigated in Fig. 8. To investigate the effect of strip gaps, the parameter *G* is varied from 30 to 80 nm. In all the simulations the strip widths are decreased to maintain the previous value of the periodicity. Based on the results of Fig. 8a gap plasmons play an important role in the wideband performance since by increasing the gap distance, the 90% frequency bandwidth becomes narrower.

**Figure 9:**
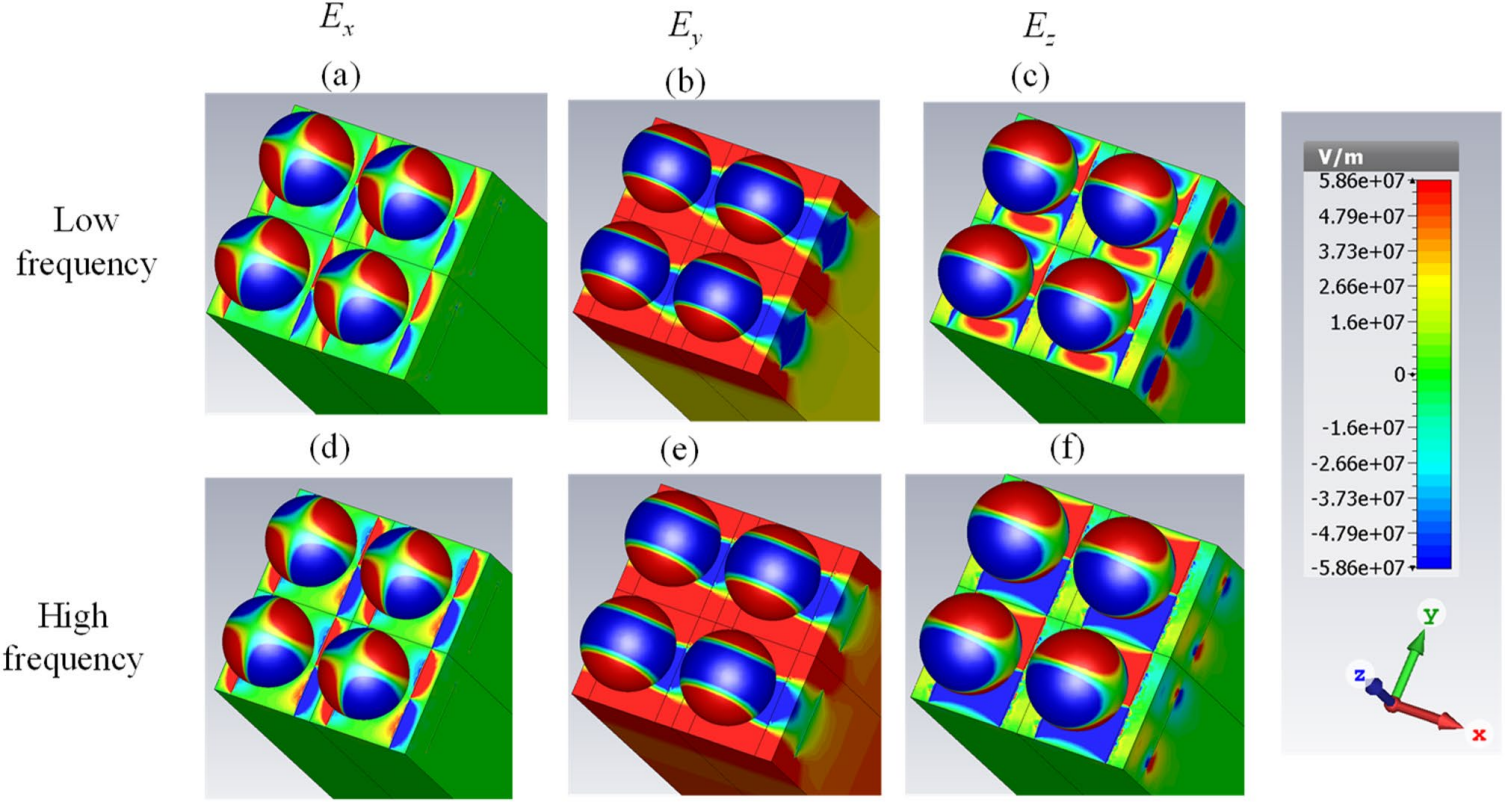
The electric field distributions for the absorber in Fig. 8a, considering *G* = 80 nm.

The electric field distributions at two resonance peaks for *G* = 80 nm is included in Fig. 9. The results indicate that the low-frequency resonance is a bulk surface plasmon resonance that is a Fabry–Perot (FP) resonance (strong field concentration in the interior region of an individual ribbon), whereas the higher-frequency resonance is an edge effect due to the proximity of graphene ribbons (high field localized between two neighboring ribbons) in the bottom meta-surface. Patterns of *E*_*z*_ in Fig. 9c,f are clarifying. The broadband nature of the absorption peak for *W* = 220 nm (corresponding to *G* = 20 nm) in Fig. 5 is caused as a contribution of the fundamental FP mode in the graphene ribbon, which is spectrally close to the edge mode, where the relatively low quality factors of both lead to an overlapping of them. Note that for every considered value of the gap parameter *G,* the absorption band coincides with the hyperbolic regime of the graphene meta-sheet, as shown in Fig. 1c. Moreover, the radii of the spheres *R* is swept from 60 to 90 nm in Fig. 8b. This modification slightly impacts the optical response.

**Figure 10:**
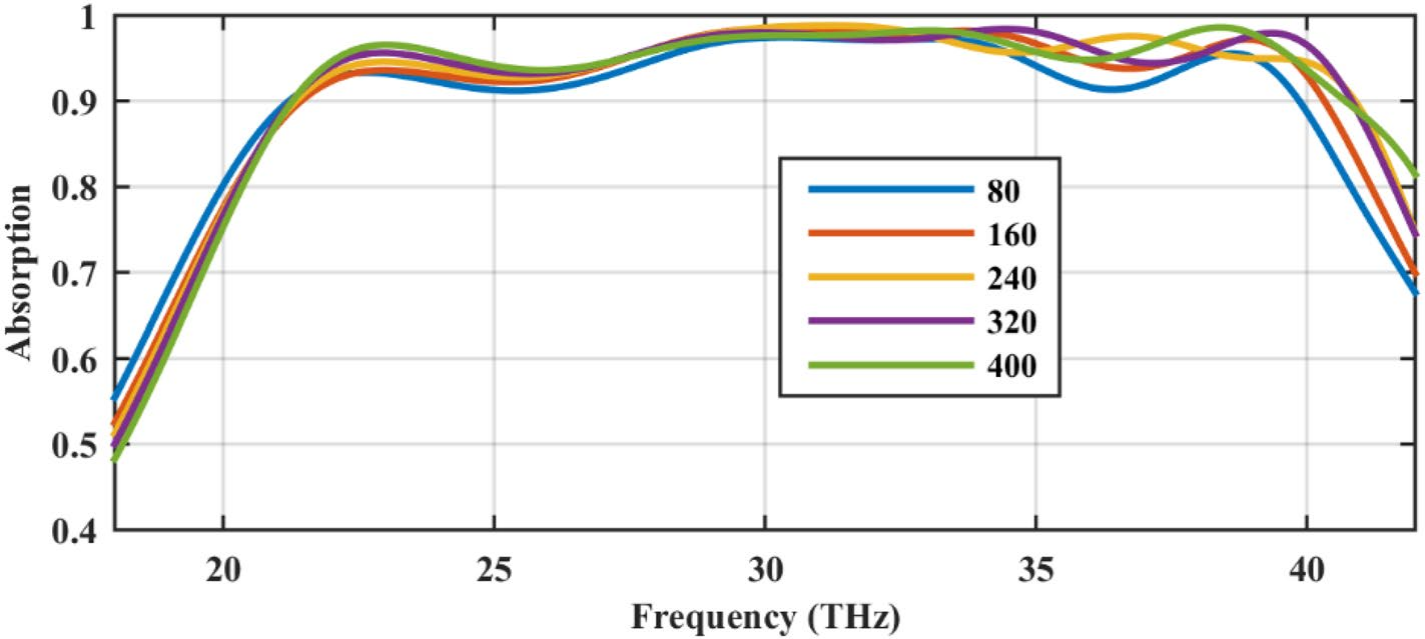
The parametric sweep of the distances between the stacked strips *d* (nm) for the chemical potential set (0.95,1.4) eV to investigate the influence of plasmonic coupling. The overall thickness of the absorber is *d* + *h* = 2 μm in all cases. The graphene-coated spheres are hollow and the relative permittivity of the substrate is 2. The geometrical parameters are *W* = 220 nm, *G* = 20 nm, and *R* = 100 nm.

Figure 10 confirms that by changing the relative distances of the strips from *d* = 80–400 nm, the performance is slightly changed, indicating that the inter-coupling of surface plasmons in graphene sheets is a neglecting mechanism for absorption, enabling a robust performance against fabrication tolerance. This performance is expected due to the extreme light confinement in the graphene-based densely packed strips^40^. Similarly, the local nature of surface plasmons has resulted in the periodicity independent behavior in another graphene-based absorber based on magnetic dipole resonances^88^. This result can be further confirmed by comparing Fig. 5b (only bottom strip) with Fig. 5c (without particles), which are almost identical. In the simulations of this figure, the overall thickness is *d* + *h* = 2 μm.

**Figure 11:**
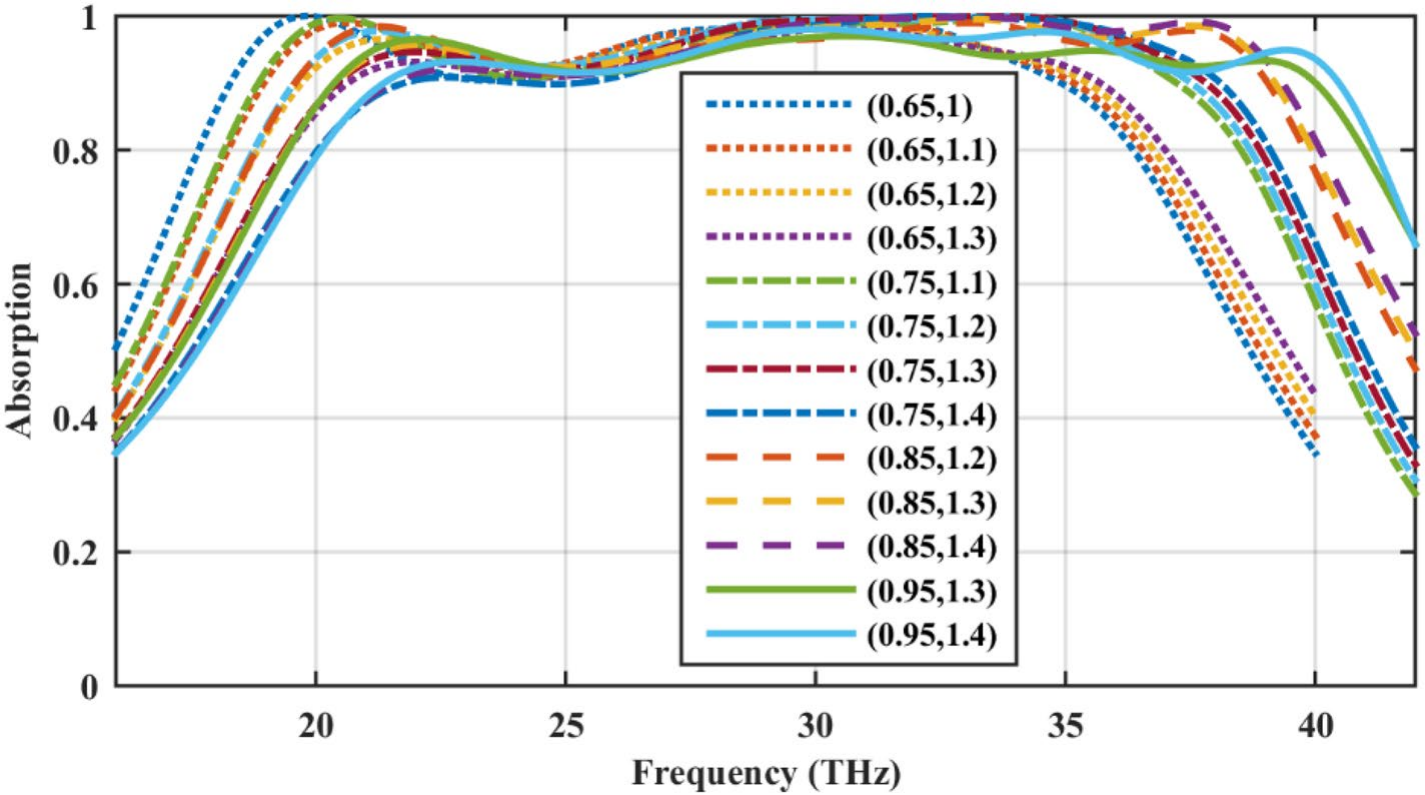
Wideband absorption for different sets of chemical potentials. The graphene-coated spheres are hollow and the relative permittivity of the substrate is 2. The geometrical parameters are *W* = 220 nm, *G* = 20 nm, *R* = 100 nm, *d* = 100 nm, and *h* = 1900 nm.

Finally, note that the chemical potential for the wideband performance in Fig. 5a was chosen based on multiple parametric sweeps. The wide bandwidth is maintained for other sets of properly chosen chemical potentials, as shown in Fig. 11. The nature of the underlying mechanism is the same for all sets. The dynamic broadband absorption with the 90% absorption rate for the frequency span ranging from 18.16 to 40.47 THz is confirmed using a two-state biasing scheme. Also, based on Ref.^89^, the maximum attainable bandwidth using a single layer of graphene-coated particles is around 7.13 THz which can be extended to 13.96 THz by stacking double layers of such particles with optimized parameters. The present design has a single layer of particles and its substrate thickness is around half of Ref.^89^ while providing 22.31 THz of dynamic bandwidth. This design offers the opportunity to the dynamic tuning of the chemical potential, as well. On the other hand, the dynamic tuning of the absorbers in Ref.^89^ are not practically feasible due to the isolation of the particles. The bandwidth can be further improved by engineering particle size to excite other plasmonic resonances^19^.

## Conclusion

By simultaneously exciting the localized surface plasmon resonances of graphene-coated nanoparticles and the propagating surface plasmons of stacked densely packed graphene strips, a wideband optical absorber can be realized. Bandwidth enhancements can be interpreted based on the anisotropic nature of tightly coupled graphene strips which behave as a 2D hyperbolic material in the desired operating bandwidth. The narrow gaps between the strips result in the excitation of the gap plasmons as another absorption enhancement mechanism. Moreover, once these anisotropic layers are stacked in the cross-shaped form, they provide a polarization-independent absorption. The proposed structure offers a simple approach for practical biasing of the graphene-based nanoparticles deposited on the strips. The real-time plasmonic performance tuning is feasible to further enhance the dynamic bandwidth of the structure. The wideband absorption bandwidth of our proposed structure is maintained for the oblique angles up to around 40° and it is lightweight and sub-wavelength.
